# Trends in Pulmonary Hypertension Mortality and Morbidity

**DOI:** 10.1155/2014/105864

**Published:** 2014-06-01

**Authors:** Alem Mehari, Orlando Valle, Richard F. Gillum

**Affiliations:** Department of Medicine, Howard University, 2041 Georgia Avenue NW, Washington, DC 20060, USA

## Abstract

*Context.* Few reports have been published regarding surveillance data for pulmonary hypertension, a debilitating and often fatal condition. *Aims.* We report trends in pulmonary hypertension. *Settings and Design.* United States of America; vital statistics, hospital data. *Methods and Material.* We used mortality data from the National Vital Statistics System (NVSS) for 1999–2008 and hospital discharge data from the National Hospital Discharge Survey (NHDS) for 1999–2009. *Statistical Analysis Used.* We present age-standardized rates. *Results.* Since 1999, the numbers of deaths and hospitalizations as well as death rates and hospitalization rates for pulmonary hypertension have increased. In 1999 death rates were higher for men than for women; however, by 2002, no differences by gender remained because of the increasing death rates among women and the declining death rates among men; after 2003 death rates for women were higher than for men. Death rates throughout the reporting period 1999–2008 were higher for blacks than for whites. Hospitalization rates in women were 1.3–1.6 times higher than in men. *Conclusions.* Pulmonary hypertension mortality and hospitalization numbers and rates increased from 1999 to 2008.

## 1. Introduction

Pulmonary hypertension (PH) is a disorder of the pulmonary vasculature that results in increased pulmonary arterial pressure and is defined as a mean pulmonary arterial pressure (mPAP) ≥25 mm Hg at rest, the pressure being measured invasively with a pulmonary artery catheter [1–4]. Despite improvements in the diagnosis and management of PH over the past 2 decades with the introduction of targeted medical therapies leading to improved survival, the disease continues to have a poor long-term prognosis [5]. US death rates for PH as the underlying cause of death increased between 1979 and 1999 [6–8]. To assess more recent trends, this report describes national trends for all PH-related deaths and hospitalizations during 1999–2008.

## 2. Subjects and Methods

To examine trends in PH mortality, we analyzed data from the Centers for Disease Control (CDC) National Vital Statistics System (NVSS). The NVSS classified diseases and conditions reported on death certificates during 1999–2010 according to the* International Classification of Diseases, Tenth Revision* (ICD-10) codes for deaths [9–19]. For this analysis, we considered PH-associated deaths, those with ICD-10 codes I27.0, I27.8, or I27.9 during 1999–2002 and ICD-10 codes I27.0, I27.2, I27.8, or I27.9 during 2003–2008, as any contributing cause of death (i.e., any of the possible 20 conditions, including underlying cause) on their death certificate. We used resident populations from the U.S. Census Bureau to calculate death rates per 100,000 population. We age-standardized death rates to 2000 U.S. standard population [16, 17]. Because the numbers of deaths with PH were relatively small each year, we aggregated years according to the availability of drugs for PH, that is, when only epoprostenol was available (1999–2001), after availability of bosentan (2002–2005), and after sildenafil entered the market (2006–2009) to produce stable statistics and shed light on the possible role of these new drugs on mortality and hospitalization.

To examine trends in PH hospitalization, we analyzed data from the Centers for Disease Control (CDC) National Hospital Discharge Survey (NHDS) for 1999–2009 [20–22]. We report estimates of all-listed diagnoses of PH (ICD-9-CM codes 416.0, 416.8, or 416.9) based on counting up to seven medical diagnoses recorded in NHDS and using sampling weights (i.e., inflation factors) that allow estimation of US statistics from the sample.

We used the U.S. civilian population for the period 1999–2009 from the U.S. Census Bureau to calculate age- and sex-specific diagnosis rates per 100,000 population. To examine trends in diagnoses during 1999–2009, we aggregated data into one 3-year and two 4-year periods (1999–2001, 2002–2005) and (2006–2009) as for mortality (see above).

Supplementary Material provides detailed tables not included in this report. (See Supplementary Tables in the Supplementary Material available online at http://dx.doi.org/10.1155/2014/105864).

## 3. Results

### 3.1. Mortality

During 1999–2008, the total number of deaths with PH listed as any contributing cause of death increased from 15,046 in 1999 to 19,373 in 2008. For the reporting periods 1999–2002 and 2003–2008, 57.7% and 61%, respectively, of decedents with PH-related mortality were female. In 1999–2002, 11.2% of males and 9.7% of females were <45 years; in 2003–2008, 8.4% of males and 6.9% of females were aged <45 years. The proportion of decedents aged ≥85 years increased from 17.6% to 22.4%. At all ages in 1999–2008, the most common underlying cause of death was pulmonary hypertension (29.4–30.8%), followed by chronic lower respiratory disease (20.2–27.0%). Among decedents aged <45 years, the most common underlying causes of death were PH; congenital malformations; complications of pregnancy, childbirth, and the puerperium; or conditions originating in the perinatal period. Supplementary Material provides further detailed mortality data in supplementary Tables 1–9.

**Figure 1:**
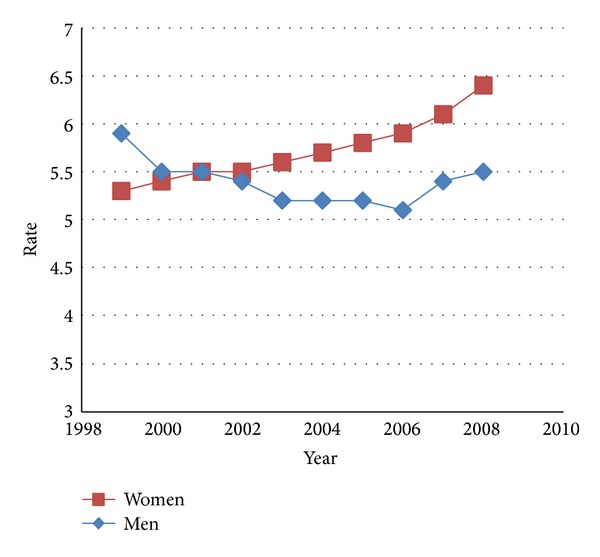
Age-standardized death rate per 100,000 population for decedents with pulmonary hypertension listed as any contributing cause of death by sex and year—United States 1999–2008. Age is standardized using the 2000 U.S. standard population.

In 1999–2008, age-standardized death rates for the total U.S. population remained relatively stable from 1999 through 2008 (Table 1). However, age-standardized death rates increased among women; among men rates decreased during 1999–2006 then increased during 2007-2008 (Figure 1). Rates increased with age. Blacks had higher rates than whites for each year in the reporting period. Non-Hispanics had higher age-standardized death rates than Hispanics. There was an upward trend in age-specific death rates for people aged ≥85 years, most markedly during 2006–2008. In 1999–2001, men had slightly higher age-specific death rates than women, but in 2002–2008 women had higher age-specific death rates than men in the older age groups. At ages <75 years, blacks had higher age-specific death rates than whites during 1999–2001, but whites had higher death rates than blacks at ages ≥85 years. States with the 10 highest age-standardized death rates were Vermont (11.4), Wyoming (10), Colorado (8.9), Idaho (7.9), DC (7.7), Montana (7.5), North Carolina (7.5), Ohio (7.5), West Virginia (7.3), and New Mexico (7.4). Supplementary Material provides further detailed hospitalization data in supplementary Tables 10–13.

### 3.2. Hospitalizations

**Figure 2:**
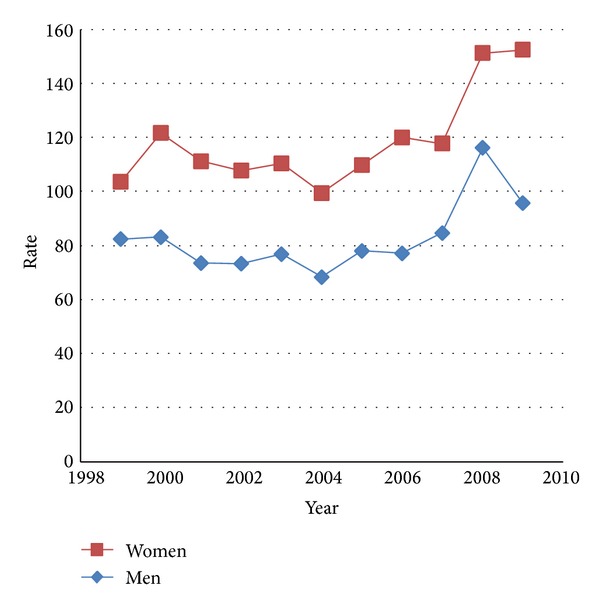
Estimated annual rate per 100,000 population of all-listed diagnoses of pulmonary hypertension by year and sex: National Hospital Discharge Survey, United States, 1999–2009.

From 1999 to 2009, the estimated number of all-listed diagnoses of PH increased by 1.5 times from 257 thousand to 386 thousand. Women accounted for 59.3–60.3% and patients aged ≥65 years accounted for 62.6–66.7% of all-listed diagnoses of PH. All-listed diagnosis rates in 2006–2009 were 1.2–1.4 times higher in men and 1.5 times higher in women than earlier (Table 2). The rate of all-listed diagnoses increased for all age groups; the greatest increase was among adults aged ≥65 years. Rates of diagnoses for PH among women were higher than those for men throughout the study period (Figure 2). Rates increased with age. Rates were highest in the northeast region.

## 4. Discussion

During 1999–2008, rates for PH as any contributing cause of death and as all-listed hospital diagnoses increased. The number of PH-related deaths and number of hospitalizations increased, particularly among women, blacks, and older adults. In addition, PH was the most common reported underlying cause of death among all decedents with PH as any contributing cause of death during 2000–2008. PH might have been diagnosed more often as diagnostic and therapeutic options improve. Increased survival of patients with PH receiving more effective therapy over the last two decades could also play a role.

The observed increases in reporting of PH as any-listed diagnosis on hospital records might indicate an actual increase in the number of patients or, more likely, a greater awareness among physicians. Increased awareness of PH as a fatal condition could lead to increased reporting of PH as a contributing cause of death [23, 24]. Even though patients with PH die due to right heart failure, heart failure is rarely reported on PH death certificates as the underlying cause because specific instructions on the death certificate state that “cardiac failure” should not be listed as the underlying cause of death.

Although the question cannot be directly addressed with these data, one may wish to consider the possible effect on PH mortality and hospitalizations of the introduction of new drugs for PH, namely, epoprostenol sodium, followed by bosentan and sildenafil to the US market. Epoprostenol (for injection) was approved for PH by the United States Food and Drug Administration (FDA) in 1995. Bosentan (oral) was approved for PH by the United States Food and Drug Administration (FDA) in 2001. Sildenafil (for injection and oral) is marketed for PH and was approved for PH by the United States Food and Drug Administration (FDA) in 2005 (Source Pulmonary Hypertension Association, http://www.phassociation.org/Treatments, last accessed 5/13/2014). These data are consistent with increased availability of approved drugs for the treatment of PH, some given by infusion, contributing to increased rates of hospitalization and listing of PH discharge diagnoses in the most recent period shown in Table 2 (2006–2009). Increasing frequency of premortem diagnoses also could have led to increased listing of PH as a contributing cause of death on death certificates leading to apparent increases in rates over the period shown in Table 1.

Our findings are similar to those of Davis et al., who suggested that annual age-adjusted mortality for idiopathic pulmonary arterial hypertension increased between 1979–1996 and 1994–1998, the greatest increase was among black women and that the disease may not be uncommon in the elderly [6, 8]. Similarly, Hyduk et al. observed increasing rates of hospitalizations for PH and mortality with PH over the period 1980–2002, particularly among women, blacks, and older adults. In their report, 30% of the patients dying with PH were 75 years of age or older [7]. In addition, age-specific death rates for PH increased among men 65 years of age or older, whites 75 years of age or older, and blacks 65 years of age or older. Similar to our report, during 1990–2002 death rates were higher for blacks than whites. The proportion of patients dying with PH who were 75 years of age or older increased from 1980 to 2002; 30.6% of all patients dying with PH from 2000 to 2002 were 75 to 84 years of age, and 18.1% were 85 years of age or older, compared with 23.7% and 6.5%, respectively, from 1980 to 1984 [7]. Hence, these results combined with our results indicate long-term trends that may be expected to continue during the remainder of this decade. It will be important to learn what portion of the trends is due to increased physician awareness and changes in diagnosis and reporting.

Estimates for prevalence of idiopathic pulmonary arterial hypertension cannot be ascertained from our report. However, registry data suggest that the average age and percent female have increased in the last two decades [25–27]. In the Registry to Evaluate Early and Long-term Pulmonary Arterial Hypertension Disease Management, mean age at diagnosis was 47 years and 78% were female. Possible explanations for the increased female predominance include increased representation in the US population of ethnic groups (e.g., blacks, Hispanics) with a higher female: male ratio in patients with PH, increased environmental or iatrogenic and hormonal influences (particularly estrogens) in the pathogenesis of PH, and the increasing prevalence of severe obesity [28]. Another possible explanation is gender difference in health seeking behavior for reporting health problems and seeking treatment. A survivor bias may also exist with a greater proportion of female patients living longer or responding better to therapy [29]. Explanations for the older age include a change in the population age distribution, in the natural history of the disease itself (e.g., a change in some unrecognized intrinsic or extrinsic factor that delays disease manifestations), improved survival with therapy, or an increased prevalence of chronic lung disease in women due to secular trends in smoking among women. Our findings of excess PH mortality in blacks are generally consistent with the association between race and excess mortality from disease of the circulatory system in the United States [30]. Mechanisms for gender and racial differences in PH occurrence have been reviewed elsewhere [30, 31].

The highest hospital diagnosis rate was observed in the Northeast United States and highest death rates for PH were observed in Vermont and in the Rocky Mountain Western United States (Colorado, Idaho, Montana, New Mexico, and Wyoming). Possible explanations for this geographic pattern include the following: first, these cases might be more likely to be detected by physicians affiliated with PH centers that are located in Denver (CO) and Philadelphia (PA). Second, altitude might also play an important role in the development of PH in states such as Colorado, Montana, and Wyoming [6–8]. Identification of these patterns warrants further investigations.

Our study has several limitations. First, mortality data are subject to diagnosis and reporting errors by physicians, medical examiners, and coroners. Second, the current ICD classifications do not allow data users to differentiate specific diseases that are associated with PH. For example, the current ICD codes do not allow differentiation of the five major categories of PH [2, 3]. Thus, population estimates for WHO group I pulmonary arterial hypertension cannot be ascertained, a category of interest to researchers. Finally, hospital discharge data cannot be used to estimate prevalence or incidence of the disease because distinguishing between first hospitalizations or readmissions during the year is not possible and some patients may not be hospitalized. However, the NVSS and NHDS databases are useful for surveillance and monitoring trends.

## Supplementary Material

Supplementary Materials provide detailed tables on mortality and hospitalization not included in this report.

## Figures and Tables

**Table 1:** Age-standardized and age-specific death rates* for pulmonary hypertension^†^ as any contributing cause of death for groups defined by selected characteristics and by period: United States, 1999–2008.

Characteristic	1999–2001	2002–2005	2006–2008
Age-standardized^††^ death rate			
All	5.5	5.5	5.8
Men	5.6	5.3	5.3
Women	5.4	5.7	6.1
Race			
Black	7.4	7.6	8.1
White	5.3	5.3	5.6
Asian/Pacific Islander	2.6	2.5	2.8
American Indian	4.0	3.9	3.9
Hispanic origin			
Hispanic	3.2	3.1	3.1
Non-Hispanic	5.6	5.7	5.9
Age-specific death rate			
0–44 yrs	0.9	0.8	0.7
45–54 yrs	2.8	2.7	2.8
55–64 yrs	7.2	7.0	7.0
65–74 yrs	19.4	19.0	18.3
75–84 yrs	37.1	39.0	43.0
>85 yrs	60.8	67.4	80.0

*Per 100,000 population; ^†^International Classification of Diseases, Tenth Revision Codes I27.0, I27.2, I27.8, and I27.9; ^††^to the 2000 U.S. standard population.

**Table 2:** Estimated rate* of pulmonary hypertension^†^ all-listed diagnoses during hospital stay, by selected characteristics and period: National Hospital Discharge Survey, United States, 1999–2009.

Characteristic	1999–2001 rate*	2002–2005 rate*	2006–2009 rate*
Total	96.3	90.8	115.1
Male	79.7	74.1	93.4
Female	112.2	106.9	135.4
15–44 years	14.1	18.3	20.2
45–64 years	103.4	104.5	100.0
65 and over	527.2	453.0	598.7
Northeast	120.8	103.4	132.6
Midwest	99.0	92.0	112.6
South	90.6	91.3	122.1
West	79.3	78.1	92.6

*Per 100,000 population; ^†^International Classification of Diseases, Ninth Revision, Clinical Modification codes 416.0, 416.8, and 416.9.
