# Supplementary material for: Trends in Pulmonary Hypertension Mortality and Morbidity
Source: Pulm Med. 2014 Jun 1;2014:105864. doi: 10.1155/2014/105864 (PMC4060165; doi:10.1155/2014/105864)
Supplement: Supplementary file 1 — Supplementary Materials provide detailed tables on mortality and hospitalization not included in this report. [file 105864.f1.docx]

**Appendix Tables**

**Table 1. Number of decedents with pulmonary hypertension* as any contributing cause of death and age-standardized death rate^†^, by sex and year – United States 1999 – 2008**

|  | | **Men** | | | **Women** | | | **Total** | |
| --- | --- | --- | --- | --- | --- | --- | --- | --- | --- |
| **Year** | **No.** | | **Rate** | **No.** | | **Rate** | **No.** | | **Rate** |
| 1999 | 6618 | | 5.9 | 8428 | | 5.3 | 15046 | | 5.5 |
| 2000 | 6273 | | 5.5 | 8629 | | 5.4 | 14902 | | 5.4 |
| 2001 | 6514 | | 5.5 | 9083 | | 5.5 | 15597 | | 5.5 |
| 2002 | 6484 | | 5.4 | 9184 | | 5.5 | 15668 | | 5.4 |
| 2003 | 6357 | | 5.2 | 9553 | | 5.6 | 15910 | | 5.5 |
| 2004 | 6525 | | 5.2 | 9861 | | 5.7 | 16386 | | 5.5 |
| 2005 | 6649 | | 5.2 | 10231 | | 5.8 | 16880 | | 5.6 |
| 2006 | 6570 | | 5.1 | 10644 | | 5.9 | 17214 | | 5.6 |
| 2007 | 7047 | | 5.4 | 11117 | | 6.1 | 18164 | | 5.7 |
| 2008 | 7400 | | 5.5 | 11973 | | 6.4 | 19373 | | 6.0 |

*International Classification of Diseases, Tenth Revision Codes I27.0, I27.2, I27.8, I27.9 ; ^†^ Per 100,000 population. Age-standardized to the 2000 U.S. standard population

**Table 2. Number of decedents with pulmonary hypertension* as any contributing cause of death and age-standardized death rate,^†^ by race and year – United States 1999 – 2008**

|  | | **White** | | | **Black** | | | **Asian/Pacific**  **Islander** | | | **American Indian** | |
| --- | --- | --- | --- | --- | --- | --- | --- | --- | --- | --- | --- | --- |
| **Year** | **No.** | | **Rate** | **No.** | | **Rate** | **No.** | | **Rate** | **No.** | | **Rate** |
| 1999 | 12740 | | 5.3 | 2043 | | 7.6 | 195 | | 2.8 | 68 | | 4.4 |
| 2000 | 12682 | | 5.2 | 1952 | | 7.3 | 200 | | 2.5 | 68 | | 4.1 |
| 2001 | 13279 | | 5.4 | 2032 | | 7.3 | 219 | | 2.6 | 67 | | 3.5 |
| 2002 | 13335 | | 5.3 | 2061 | | 7.3 | 203 | | 2.2 | 69 | | 3.5 |
| 2003 | 13506 | | 5.3 | 2079 | | 7.4 | 250 | | 2.6 | 75 | | 3.9 |
| 2004 | 13822 | | 5.3 | 2222 | | 7.6 | 254 | | 2.6 | 88 | | 4.1 |
| 2005 | 14225 | | 5.4 | 2316 | | 7.9 | 249 | | 2.4 | 90 | | 4.1 |
| 2006 | 14499 | | 5.4 | 2360 | | 7.8 | 262 | | 2.5 | 93 | | 4.2 |
| 2007 | 15266 | | 5.6 | 2502 | | 8.2 | 309 | | 2.8 | 87 | | 3.7 |
| 2008 | 16333 | | 5.8 | 2603 | | 8.3 | 347 | | 3.0 | 90 | | 3.7 |

*International Classification of Diseases, Tenth Revision Codes I27.0, I27.2, I27.8, I27.9; ^†^ Per 100,000 population. Age-standardized to the 2000 U.S. standard population

**Table 3. Number of decedents with pulmonary hypertension* as any contributing cause of death and age-standardized death rate, ^†^ by Ethnicity and year – United States, 1999 – 2008**

|  | | **Hispanic** | | | **Non-Hispanic** | |
| --- | --- | --- | --- | --- | --- | --- |
| **Year** | **No.** | | **Rate** | **No.** | | **Rate** |
|  |  | |  |  | |  |
| 1999 | 552 | | 3.0 | 14447 | | 5.6 |
| 2000 | 625 | | 3.4 | 14234 | | 5.5 |
| 2001 | 645 | | 3.1 | 14916 | | 5.6 |
| 2002 | 617 | | 3.0 | 15003 | | 5.6 |
| 2003 | 651 | | 3.1 | 15222 | | 5.6 |
| 2004 | 735 | | 3.1 | 15630 | | 5.7 |
| 2005 | 739 | | 3.0 | 16118 | | 5.7 |
| 2006 | 754 | | 3.1 | 16431 | | 5.7 |
| 2007 | 777 | | 2.9 | 17364 | | 5.9 |
| 2008 | 865 | | 3.2 | 18483 | | 6.2 |

*International Classification of Diseases, Tenth Revision Codes I27.0, I27.2, I27.8, I27.9; ^†^Per 100,000 population. Age-standardized to the 2000 U.S. standard population.

**Table 4. Number of decedents with pulmonary hypertension* as any contributing cause of death and age-specific death rate,^†^ by age group and year – United States, 1999 – 2008**

|  | **0 - 44 yrs.** | | **45 - 54 yrs.** | | **55 - 64 yrs.** | | **65 - 74 yrs.** | | **75 - 84 yrs.** | | **> 85 yrs.** | |
| --- | --- | --- | --- | --- | --- | --- | --- | --- | --- | --- | --- | --- |
| **Year** | **No.** | **Rate** | **No.** | **Rate** | **No.** | **Rate** | **No.** | **Rate** | **No.** | **Rate** | **No.** | **Rate** |
| 1999 | 1630 | 0.9 | 1014 | 2.8 | 1779 | 7.5 | 3677 | 20.0 | 4519 | 37.0 | 2427 | 58.4 |
| 2000 | 1535 | 0.8 | 1047 | 2.8 | 1726 | 7.1 | 3511 | 19.1 | 4522 | 36.6 | 2561 | 60.4 |
| 2001 | 1635 | 0.9 | 1081 | 2.8 | 1793 | 7.1 | 3503 | 19.1 | 4755 | 37.8 | 2829 | 63.6 |
| 2002 | 1555 | 0.8 | 1122 | 2.8 | 1848 | 6.9 | 3343 | 18.3 | 4830 | 37.9 | 2969 | 64.6 |
| 2003 | 1324 | 0.7 | 1055 | 2.6 | 1950 | 7.0 | 3426 | 18.7 | 5044 | 39.2 | 3111 | 66.0 |
| 2004 | 1506 | 0.8 | 1184 | 2.8 | 2039 | 7.0 | 3430 | 18.6 | 4921 | 37.9 | 3305 | 68.0 |
| 2005 | 1307 | 0.7 | 1166 | 2.7 | 2120 | 7.0 | 3466 | 18.6 | 5207 | 39.9 | 3614 | 70.9 |
| 2006 | 1219 | 0.7 | 1210 | 2.8 | 2179 | 6.9 | 3401 | 18.0 | 5279 | 40.5 | 3926 | 74.1 |
| 2007 | 1208 | 0.6 | 1212 | 2.8 | 2331 | 7.1 | 3538 | 18.3 | 5482 | 42.1 | 4393 | 79.7 |
| 2008 | 1307 | 0.7 | 1233 | 2.8 | 2325 | 6.9 | 3717 | 18.5 | 5873 | 45.1 | 4918 | 86.0 |

*International Classification of Diseases, Tenth Revision Codes I27.0, I27.2, I27.8, I27.9; ^†^Per 100,000 population. Age-standardized to the 2000 U.S. standard population.

**Table 5. Age-standardized and age-specific death rates* for pulmonary hypertension† as anycontributing cause of death for groups defined by selected characteristics, by period – United States, 1999 – 2008**

| **Characteristic** | **1999 - 2002** | **2003 - 2008** |
| --- | --- | --- |
| **Age-Standardized**^††^**Death Rate** |  |  |
| All | 5.4 | 5.7 |
| Men | 5.6 | 5.3 |
| Women | 5.4 | 5.9 |
| Race |  |  |
| Black | 7.4 | 7.8 |
| White | 5.3 | 5.5 |
| Asian/Pacific Islander | 2.5 | 2.6 |
| American Indian | 3.8 | 3.9 |
| Hispanic Origin |  |  |
| Hispanic | 3.1 | 3.1 |
| Non-Hispanic | 5.6 | 5.8 |
| **Age-Specific Death Rate** |  |  |
| All |  |  |
| 0 - 44 yrs | 0.9 | 0.7 |
| 45 - 54 yrs | 2.8 | 2.8 |
| 55 - 64 yrs | 7.1 | 7.0 |
| 65 - 74 yrs | 19.1 | 18.4 |
| 75 - 84 yrs | 37.3 | 40.8 |
| > 85 yrs | 61.9 | 74.6 |

*Per 100,000 population; ^†^International Classification of Diseases, Tenth Revision Codes I27.0, I27.2, I27.8, I27.9; ^††^to the 2000 U.S. standard population

**Table 6. Age--specific death rates* for pulmonary hypertension† as any contributing cause of death for age groups defined by gender and race , by period – United States, 1999 – 2008**

| **Age-Specific Death Rate** | **1999 - 2002** | **2003 - 2008** |
| --- | --- | --- |
| **Men** |  |  |
| 0 - 44 yrs | 0.8 | 0.6 |
| 45 - 54 yrs | 2.4 | 2.4 |
| 55 - 64 yrs | 6.6 | 6.1 |
| 65 - 74 yrs | 19.4 | 17.2 |
| 75 - 84 yrs | 40.3 | 39.9 |
| > 85 yrs | 69.5 | 71.1 |
| **Women** |  |  |
| 0 - 44 yrs | 0.9 | 0.8 |
| 45 - 54 yrs | 3.1 | 3.1 |
| 55 - 64 yrs | 7.6 | 7.8 |
| 65 - 74 yrs | 18.9 | 19.4 |
| 75 - 84 yrs | 35.3 | 41.4 |
| > 85 yrs | 58.7 | 76.2 |
| **Whites** |  |  |
| 0 - 44 yrs | 0.7 | 0.6 |
| 45 - 54 yrs | 2.4 | 2.3 |
| 55 - 64 yrs | 6.7 | 6.4 |
| 65 - 74 yrs | 18.9 | 18.1 |
| 75 - 84 yrs | 37.8 | 41.2 |
| > 85 yrs | 63.1 | 76.4 |
| **Blacks** |  |  |
| 0 - 44 yrs | 1.7 | 1.5 |
| 45 - 54 yrs | 6.2 | 6.3 |
| 55 - 64 yrs | 13.0 | 13.5 |
| 65 - 74 yrs | 25.5 | 26.4 |
| 75 - 84 yrs | 38.3 | 45.4 |
| > 85 yrs | 55.6 | 67.1 |

*Per 100,000 population; ^†^International Classification of Diseases, Tenth Revision Codes I27.0, I27.2, I27.8, I27.9; ^††^to the 2000 U.S. standard population

**Table 7. Percentage of selected characteristics among decedents with pulmonary hypertension* reported as any contributing cause of death, by period – United States, 1999 – 2008**

| **Characteristic** | **1999 – 2002 (N=61,213)** | **2003-2008 (N=103,9270)** |
| --- | --- | --- |
|  |  |  |
| Women | 57.7 | 61.0 |
| Men | 42.3 | 39.0 |
| Race |  |  |
| White | 85.0 | 84.3 |
| Black | 13.2 | 13.5 |
| Other race | 1.8 | 2.2 |
| Hispanic Origin | 4.0 | 4.4 |
| Non-Hispanic | 95.7 | 95.5 |
| Age Groups |  |  |
| 0- 44 yrs | 10.4 | 7.6 |
| 45 - 54 yrs | 7.0 | 6.8 |
| 55 - 64 yrs | 11.7 | 12.5 |
| 65 - 74 yrs | 22.9 | 20.2 |
| 75 - 84 yrs | 30.4 | 30.6 |
| >85 yrs | 17.6 | 22.4 |

*International Classification of Diseases, Tenth Revision Codes I27.0, I27.2, I27.8, I27.9

**Table 8. Percentage of selected causes of death reported as the underlying cause of death among all decedents with pulmonary hypertension reported as any contributing cause of death, by period –United States, 1999-2008**

| **Underlying cause of death reported on death certificate (ICD -10 codes*** | **1999-2002**  **(N=61,213)** | **2003-2005**  **(N=49,176)** | **2006-2008**  **(N=54,751)** |
| --- | --- | --- | --- |
| Pulmonary hypertension (I27.0, I27.2, I27.8, I27.9) | 29.5 | 30.8 | 29.4 |
| Chronic lower respiratory diseases J40-J47) | 27.0 | 22.1 | 20.2 |
| Ischemic heart disease(I20-I25) | 8.6 | 9.8 | 10.2 |
| All other cardiovascular diseases(I00-I19,I26,I27.1,I28-I34,I40-I49,I51-I78) | 8.0 | 8.4 | 8.5 |
| All other respiratory diseases (J00-J06,J20-J22, J60-J98) | 5.8 | 6.1 | 6.5 |
| Congenital malformations(Q00-Q99) | 2.9 | 2.3 | 1.9 |
| Chronic valvular heart disease(I35-I39) | 1.9 | 2.2 | 2.7 |
| Malignant neoplasm of trachea, bronchus, and lung( C33-C44) | 1.8 | 1.5 | 1.3 |
| Systemic lupus erythromatosis (M32), Systemic sclerosis(M34), Dermatomyositis(M33), Sicca syndrome(M35), Rheumatoid arthritis(M05-M06), and Juvenile arthritis(M08) | 1.4 | 2.5 | 2.6 |
| Complications of pregnancy, childbirth, and the puerperium (O10-O99), or conditions originating in the perinatal period(P00-P96) | 1.1 | 0.6 | 0.4 |
| Influenza and pneumonia(J10-J18) | 0.9 | 1.2 | 1.2 |
| Heart Failure (I50) | 0.7 | 0.6 | 0.6 |
| Chronic liver disease and cirrhosis(K70, K73-K74) | 0.3 | 0.4 | 0.6 |
| Human immunodeficiency Virus infection (O42-O44/B20-B24) | 0.1 | 0.1 | 0.2 |
| All other causes | 12.5 | 13.9 | 15.3 |

*International classification of Diseases, Tenth Revision (ICD-10) codesI27.0, I27.2, I27.8, I27.9

**Table 9. Percentage of selected causes of death reported as the underlying cause of death among decedents aged<45 years with pulmonary hypertension reported as any contributing cause of death , by period –united states, 2000-2008**

| **Underlying cause of death reported on death certificate (ICD- 10codes*)** | **1999-2002**  **(N=6,355)** | **2003-2005**  **(N=4,137)** | **2006-2008**  **(N=3,734)** |
| --- | --- | --- | --- |
|  |  |  |  |
| Pulmonary hypertension (I27.0, I27.2, I27.8, I27.9) | 40.5 | 39.6 | 35.8 |
| Congenital malformations, deformities, and chromosomal abnormalities(Q00-Q99) | 20.7 | 19.0 | 18.6 |
| Complications of pregnancy, childbirth, and the puerperium (O10-O99), or conditions originating in the perinatal period(P00-P96) | 10.8 | 7.5 | 6.2 |
| All cardiovascular diseases, excluding pulmonary hypertension( I00-I26, I27.1,I28-I78) | 6.3 | 6.3 | 7.1 |
| All other respiratory diseases ( J00-J06,J20-J22, J60-J98 | 4.0 | 5.1 | 7.2 |
| Chronic lower respiratory diseases(J40-J47) | 2.6 | 2.8 | 2.3 |
| Systemic lupus erythromatosis (M32), Systemic sclerosis  ( M34), Dermatomyositis (M33), Sicca syndrome(M35), Rheumatoid arthritis (M05-M06), and Juvenile arthritis (M08-M08) | 2.5 | 4.0 | 4.2 |
| Influenza and pneumonia(J10-J18) | 0.6 | 1.1 | 1.2 |
| All other causes | 1.0 | 12.8 | 14.7 |

*International classification of Diseases, Tenth Revision (ICD-10) codesI27.0, I27.2, I27.8, I27.9.

**Table 10.Estimated annual number and rate* of hospitalizations for persons with pulmonary hypertension† as any –listed diagnosis during hospital stay, by year and sex-national Hospital Discharge Survey, United States, 1999-2009**

|  | **Men** |  | **Women** |  | **Total** |  |
| --- | --- | --- | --- | --- | --- | --- |
|  |  |  |  |  |  |  |
| **Year** | **No** | **Rate** | **No** | **Rate** | **No.** | **Rate** |
|  |  |  |  |  |  |  |
| 1999 | 111,000 | 82.4 | 146,000 | 103.6 | 257,000 | 93.3 |
| 2000 | 113,000 | 83.1 | 173,000 | 121.7 | 286,000 | 102.8 |
| 2001 | 102,000 | 73.5 | 161,000 | 111.2 | 263,000 | 92.7 |
| 2002 | 103,000 | 73.3 | 158,000 | 107.8 | 261,000 | 90.9 |
| 2003 | 109,000 | 76.8 | 163,000 | 110.4 | 272,000 | 93.9 |
| 2004 | 98,000 | 68.3 | 148,000 | 99.4 | 246,000 | 84.1 |
| 2005 | 113,000 | 78 | 165,000 | 109.8 | 278,000 | 94.2 |
| 2006 | 113,000 | 77.1 | 182,000 | 120 | 295,000 | 98.9 |
| 2007 | 125,000 | 84.6 | 180,000 | 117.8 | 305,000 | 101.5 |
| 2008 | 173,000 | 116.2 | 233,000 | 151.3 | 405,000 | 133.7 |
| 2009 | 144,000 | 95.7 | 237,000 | 152.5 | 386,000 | 126.2 |

*Per 100,000 population.† International Classification of Diseases, Ninth Revision, Clinical Modification codes 416.0, 416.8, and 416.9

**Table 11.Estimated annual number and rate* of hospitalizations for persons with pulmonary hypertension† as any –listed diagnosis during hospital stay, by year and age group-National Hospital Discharge Survey, United States, 1999-2009**

|  | **15-44yrs** | | **45-64yrs** | | **≥65 yrs** | |
| --- | --- | --- | --- | --- | --- | --- |
| **Year** | **No.** | **Rate** | **No.** | **Rate** | **No.** | **Rate** |
|  |  |  |  |  |  |  |
| 1999 | 14,000 | 11.5 | 59,000 | 100 | 176,000 | 514 |
| 2000 | 17,000 | 13.9 | 62,000 | 101.7 | 203,000 | 588.8 |
| 2001 | 21,000 | 17 | 70,000 | 108.6 | 169,000 | 478.9 |
| 2002 | 24,000 | 19.3 | 69,000 | 103.5 | 164,000 | 460.6 |
| 2003 | 18,000 | 14.5 | 79,000 | 115.1 | 171,000 | 476.1 |
| 2004 | 23,000 | 18.5 | 69,000 | 97.7 | 148,000 | 407.8 |
| 2005 | 26,000 | 20.8 | 74,000 | 101.7 | 172,000 | 467.5 |
| 2006 | 27,000 | 21.5 | 69,000 | 55 | 192,000 | 515.3 |
| 2007 | 29,000 | 23.2 | 74,000 | 96.7 | 198,000 | 522.6 |
| 2008 | 22,000 | 17.6 | 103,000 | 132.1 | 273,000 | 702.3 |
| 2009 | 23,000 | 18.4 | 92,000 | 116 | 259,000 | 654.5 |

*Per 100,000 population.† International Classification of Diseases, Ninth Revision, Clinical Modification codes 416.0, 416.8, and 416.9

**Table 12.Estimated rate* of hospitalizations for groups with pulmonary hypertension† as any –listed diagnosis during hospital stay, by selected characteristics and period-National Hospital Discharge Survey, United States, 1999-2009.**

| **Characteristic** | **1999-2002**  **Rate*** | **2003-2006**  **Rate*** | **2007-2009**  **Rate*** |
| --- | --- | --- | --- |
| Total | 94.9 | 92.8 | 120.5 |
| Men | 78.1 | 75.0 | 98.8 |
| Women | 111.1 | 109.9 | 140.6 |
| 15-44yrs | 15.4 | 18.8 | 19.7 |
| 45-64yrs | 103.5 | 92.3 | 114.9 |
| ≥65rs | 510.6 | 466.7 | 626.5 |
| North east | 118.8 | 104.5 | 137.8 |
| Midwest | 97.7 | 92.4 | 118.3 |
| South | 89.4 | 94.5 | 129.9 |
| West | 78.6 | 81.0 | 94.0 |

*Per 100,000 population; ^†^ International Classification of Diseases, Ninth Revision, Clinical Modification codes 416.0, 416.8, and 416.9

**Table 13. Percentage of persons with selected characteristics hospitalized with pulmonary hypertension^†^ as any –listed diagnosis, by period, United States, 1999-2009.**

| **Characteristic** | **1999-2002**  **(N= 1,067,000)**  **(%)** | **2003-2006**  **(N= 1,091,000)**  **(%)** | **2007-2009**  **(N= 1,096,000)**  **(%)** |
| --- | --- | --- | --- |
| Men | 40.2 | 39.7 | 40.3 |
| Women | 59.8 | 60.3 | 59.3 |
| 15-44yrs | 7.1 | 8.6 | 6.8 |
| 45-64yrs | 24.4 | 26.7 | 24.5 |
| ≥65rs | 66.7 | 62.6 | 66.6 |
| North east | 23.6 | 20.9 | 20.7 |
| Midwest | 23.5 | 22.3 | 21.5 |
| South | 33.5 | 36.8 | 39.6 |
| West | 18.7 | 20.1 | 18.2 |

† International Classification of Diseases, Ninth Revision, Clinical Modification codes 416.0, 416.8, and 416.9 .

**Additional File**

**Additional File 1. XLS. Annual number of decedents with pulmonary hypertension* as any contributing cause of death, aggregated number, and age-standardized death rate, † by state/area – United States, 2000 – 2008**

•*International Classification of Diseases, Tenth Revision Codes I27.0, I27.2, I27.8, I27.9 reported as any contributing cause of death

•^†^ Per 100,000 population. Age-standardized to the 2000 U.S. standard population
